# Top-ten tips for managing nutritional issues and gastrointestinal symptoms in children with neurological impairment

**DOI:** 10.1186/s13052-020-0800-1

**Published:** 2020-03-27

**Authors:** Valeria Dipasquale, Frederic Gottrand, Peter B. Sullivan, Claudio Romano

**Affiliations:** 1 Department of Human Pathology in Adulthood and Childhood “G. Barresi”, Pediatric Gastroenterology and Cystic Fibrosis Unit, University of Messina, Via Consolare Valeria, 98124 Messina, Italy; 2 Department of Pediatric Gastroenterology, Hepatology and Nutrition, CHU Lille, Lille, France; 3 Department of Paediatrics, University of Oxford, Oxford, UK

**Keywords:** Neurological impairment, Undernutrition, Nutritional status, Nutritional requirement, Gastroesophageal reflux, Constipation, Enteral nutrition, Gastrostomy, Jejunal feeding, Pediatrics

## Abstract

The prevalence of children with neurological impairment (NI) presenting feeding difficulties and gastrointestinal symptoms is rising. The most recent guidelines recommend early nutritional assessment and intervention in order to prevent undernutrition and growth failure, along with the proper diagnosis and treatment of some frequent gastrointestinal symptoms, such as gastroesophageal reflux disease (GERD) and constipation, which can further worsen the feeding process and nutritional status. Nonetheless, the nutritional issues and growth deficits of children with NI are often considered to be of low priority or under recognised by healthcare providers. The present article proposes ten top tips that highlight the major points along the nutritional management pathway of NI children. The implementation of these tips in all healthcare settings could potentially improve patient outcomes and reduce morbidity and mortality.

## Introduction

Children with neurological impairment (NI) have feeding problems and gastrointestinal symptoms potentially resulting in undernutrition and growth failure, with reduced health-related quality of life. Only in the last couple of decades the nutritional needs of children with NI have become a matter of interest of many scientific research groups worldwide. In 2017, the European Society of Gastroenterology, Hepatology, and Nutrition (ESPGHAN) published a consensus statement (ESPGHAN guidelines) on the diagnosis and management of gastrointestinal and nutritional complications in children with neurological disability [1], based upon a systematic review of medical literature and experts’ opinion. The number of these children is estimated not to decline in the next years, and their life expectancy to increase [2]. Nonetheless, the nutritional issues and growth deficits in this group of children are often considered to be of low priority or under recognised by healthcare providers [2]. Therefore, focusing on their nutritional concerns is a crucial part of their overall clinical management. The present article proposes ten top tips highlighting the major areas of knowledge and intervention along the nutritional management pathway of NI children (Table 1).

**Table 1:** Top-ten tips for the nutritional management of children with neurological impairment

Item	Tip
1. Burden of NI	• Awareness of rising prevalence of NI-associated feeding difficulties and gastrointestinal symptoms
2. Assessment of nutritional status	• Standard anthropometrics and evaluation of body composition
3. Definition of undernutrition	• Physical examination and nutritional status (see Tip 2)
4. Nutritional needs	• Dietary reference intake for basal energy expenditure for typically-developing children, individualized according to motor function, muscle tone, and activity level • Daily supplements in specific clinical conditions (see Text)
5. GERD	• Early assessment and treatment • PPIs as the first line treatment
6. Constipation	• Careful history, abdominal, perineal, and eventually digital rectal examination • Osmotic agents (polyethylene glycol)
7. Enteral nutrition	• Consider start before the development of undernutrition
8. Enteral access and feeding regimen	• Gastrostomy as the preferred way for prolonged enteral tube feeding • Post-pyloric feeding in case of contraindication to gastric feeding (see Text)
9. Enteral formula	• Choice of enteral formula based on patient’s age, nutritional needs and enteral access • Safety concerns about blenderized food
10. Benefits of enteral tube feeding	• Long-term improvement of nutritional status, health-related quality of life • Low rates of serious complications

*NI* Neurological impairment, *GERD* gastroesophageal reflux disease, *PPIs* proton pump inhibitors

## Top-ten tips

- Tip 1: Be aware of the burden of neurological impairment

Neurological impairment refers to any disorder of the central nervous system that affect speech, motor skills, vision, memory and learning abilities [1]. It includes genetic and metabolic disorders, degenerative neurological disorders, prematurity, autism spectrum disorders, acquired brain injury. The major subgroup is represented by cerebral palsy (CP), such as a group of early-onset, permanent disorders that affect movement and muscle tone or posture, due to nonprogressive damages occurred in the developing fetal or infant brain [1, 3]. Cerebral palsy is one of the most frequent causes of childhood physical disability, with an estimated lifetime cost, for persons born in the United States in 2000, of 11.5 billion dollars [4]. Recent prevalence data reported 2.11 patients with cerebral palsy per 1000 live births and as high as 59.18 per 1000 live births among neonates weighing less than 1500 g [5]. Notably, despite the survival of preterm neonates has increased, the proportion with severe disability is the same [2, 6]. Different factors may explain the increased prevalence of NI, such as higher numbers of multiple births, which often result in preterm births, and a gradual improvement of life expectancy [2], with survival curves for 10-year old boys with CP showing a 50% survival into their late 20s (http://www.lifeexpectancy.org/survival.shtml). As a consequence of the increased life expectancy of NI children, the rate of feeding difficulties and gastrointestinal complaints also increased. Feeding difficulties involve up to 85% of children with NI and up to three quarters of such children are stunted [7, 8]. Moreover, nearly all children with NI have one or more comorbidity, including epilepsy (associated with long-term pharmacotherapy), muscular dystrophy and myopathies [9], which negatively affect feeding, by impeding psychomotor development and worsening symptoms like drooling and gastroesophageal reflux.

- Tip 2: Know how to assess nutritional status: standard anthropometrics and body composition

Assessment of nutritional status is the first step in the clinical nutritional evaluation of children with NI. In children with NI, measurement can be difficult and the references commonly used in pediatric patients tend to misinterpret undernutrition. Whenever possible, weight measurement should be obtained on a digital scale or, if the child is unable to stand, on a wheelchair scale [1]. Standing height or supine length can be used in children who can stand or lay down straight. However, accurate evaluation of stature may not be possible because of spasticity, joint contractures or scoliosis. In children who are unable to stand upright due to skeletal deformity, alternative measurements for the height assessment should be segmental lengths, such as knee-heel length, tibia length, and ulnar length, assessed by sliding callipers [1, 10]. Special equations or charts can then be used to calculate the standing height (Table 2) [11].

**Table 2:** Equations to predict length in children with neurological impairment (adapted from Haapala et al. [11])

Age 0–12 years (Stevenson 1995)	• Estimated height: = (2.68 x knee height) + 24.2 • Estimated height = (3.26 x tibia length) + 30.8
Age ≥ 7 years (Gauld et al. 2004)	• Estimated height = 2.423 knee height + 1.327 age + 21.818 (Males) • Estimated height = 2.758 tibia lenght + 1.717 age + 36.509 (Males) • Estimated height = 2.473 knee height + 1.187 age + 21.151 (Females) • Estimated height = 2.771tibia lenght + 1.457 age + 37.748 (Females)

The anthropometric measurements have proper value if plotted into adequate growth charts. Several NI-specific growth charts containing estimated weight-for-age or height-for-age percentiles have been created. Most recently, Brooks et al. [12] developed clinical growth charts for NI children, stratified according to gender and gross motor function in accordance with the Gross Motor Function Classification System (GMFCS) [13]. However, these charts are descriptive only and not reference standards, since they show how children with NI are growing, not necessarily how they should grow. Therefore, the use of growth charts for typically-developing children to assess growth in children with NI is recommended [1].

Assessment of nutritional status in NI children should not be based on weight and height measurements alone, but should include the evaluation of body composition [1, 9, 10]. The three most commonly used measurements to calculate growth charts in typically-developing children, such as weight-to-height ratio, height for-age, and weight-for-age are poor predictors of body composition in this group of patients [1]. Weight measurements do not distinguish between muscle and fat mass percentages. NI children have higher fat percentages and lower lean masses than typically-developing children. Body mass index is not recommended especially when derivative measures of body length are used [1]. A misinterpretation of low body mass index values or low weight z scores may lead to overfeeding, particularly in children receiving enteral tube feeding [14]. The most reliable parameters to define nutritional status in children with NI are skinfold thickness measurements and bioelectric impedance analysis [1, 15, 16]. Skinfold thickness measurements, primarily the triceps and subscapular skinfold thicknesses, are the most cost-effective and widely-available methods to assess body composition [1]. The results can then be evaluated by different equations, mainly the Slaughter equation for typically-developing children [15]. Though, these equations do not take into account the different body compositions of children with CP, in whom more body fat is stored more centrally (abdomen) than peripherally (skinfolds) and, consequently, their total body fat percentage may be underestimated. In order to improve the accuracy of the Slaughter equation, Gurka et al. [16] developed coefficients that correct for sex, race, size, pubertal status, and GMFCS level. Bioelectric impedance analysis may be more reliable than skinfold thickness in the estimation of body fat percentage as it estimates body composition regardless of fat localization [17]. Whole-body dual-energy x-ray absorptiometry (DXA) can detect typical body composition alterations in NI children, such as increased total body water, decreased fat-free mass, and decreased bone mineral density [18]. DXA scanning is the reference method to assess body composition, but it can often be challenging in children with CP due to severe scoliosis, joints contractures, and poor positioning [1]. Moreover, it is not always easy to perform, it is expensive and time-consuming, and requires specialized equipment. DXA is also recommended to measure bone mineral density as part of nutritional assessment in children with neurological disability [1].

- Tip 3: Know how to identify undernutrition

No single, universally accepted definition of undernutrition in children exists, neither in typically developing nor in neurologically impaired children [19, 20]. For clinical practice, ESPGHAN recommends the use of 1 or more red flag warning signs, including physical signs of undernutrition, such as pressure sores and poor peripheral circulation, weight for age z score < − 2, triceps skinfold thickness < 10th centile for age and sex, mid-upper arm circumference or muscle area < 10th percentile, faltering weight and/or failure to thrive [1]. These recommendations are mainly based on experts’ opinion and low/moderate-quality studies. Recently, they have been validated some screening tools aimed to identify risk of feeding and swallowing difficulties or undernutrition earlier, and to assess if further assessment is needed [21].

- Tip 4: Know how to estimate nutritional needs: calories, proteins, fluids, micronutrients

Nutritional needs are difficult to define in children with NI because of the heterogeneity of clinical aspects. Neurological impairment varies greatly, and therefore, nutritional requirements cannot be generalized. Several predictive equations are used to estimate energy needs in typically-developing children, but they are based on calories per kilogram of body weight and this may lead to an overestimation of energy requirements in children with NI [17]. Moreover, energy requirements are influenced by the severity of NI, level of physical activity, altered body composition, severity of undernutrition, or type of paralysis. Children dependent on a wheelchair require only 60 to 70% of the energy that typically- developing children require [1, 17] while those who can walk or who have athetosis have higher energy needs [1, 17]. Overestimation of energy requirements can lead to overfeeding, fat accumulation, and overweight (as is observed in 10–15% of cases) [17]. According to ESPGHAN guidelines, nutritional needs (calories, proteins, vitamins, minerals and trace elements) of children with NI can be calculated by using the dietary reference intake for basal energy expenditure for typically-developing children, then individualized by taking into account motor function, muscle tone, and activity level [1]. Indirect calorimetry (Krick method) [22] may also be used in clinical practice, but might be difficult, time-consuming, or not available. Daily supplements can be necessary in specific clinical situations, for instance the use of supplementary protein intake in case of decubitus ulcers or low energy requirement, or a daily supplement of vitamin D (about 800–1000 International Units) to prevent the deficiency secondary to antiepileptic drug use or to counteract the osteopenia secondary to immobility [1, 23]. For severely undernourished children, an intake of 2.0 g/kg per day of protein and an additional 20% increase in energy intake should be sufficient [1]. Ongoing monitoring of whether the patient is meeting the nutritional requirements is necessary to prevent over- or underfeeding. Monitoring should be based on changes in weight gain and not only on reported dietary intake, which are often inaccurate. It is recommended to check follow-up anthropometrics (weight, linear growth, and fat mass) at least every 6 months, and micronutrient levels (including vitamin D, iron status, calcium, and phosphorus) at least once a year [1].

- Tip 5: Know how to diagnose and treat gastroesophageal reflux disease

Gastroesophageal reflux disease (GERD) is one of the most frequently (up to 77%) reported gastrointestinal symptoms in children with NI, much more prevalent in NI children than in typically-developing ones [1, 24]. Despite its high prevalence, GERD in NI children is difficult to be recognized as many patients cannot express their symptoms, and symptoms are often non-specific (unexplained irritability, food rejection, hypersalivation) or atypical (anemia, increased dystonia, seizures, laryngospasm, or recurrent pulmonary infections), and associated with other complications (inhalation, swallowing difficulties) [24]. Children with NI have multiple, often coexistent risk factors for chronic, severe GERD, including decreased lower esophageal sphincter tone, delayed gastric emptying, impaired esophageal motility, chronic supine position, constipation, recurrent seizures, scoliosis, and drugs [1, 24]. Recently additional risk factors have been recognized, such as early-onset NI, abnormal electroencephalogram, and mitochondrial disease [25]. For all these reasons, children with NI should be assessed early for GERD and started with appropriate treatment in order to prevent GERD-related long-term complications. Clinical diagnosis and, whenever possible, esophagogastroduodenoscopy with biopsies and combined multiple intraluminal impedance and pH monitoring are recommended [1, 24]. Scintigraphy/swallow study could be considered in children with chronic respiratory symptoms or suspected dysphagia to evaluate for aspiration and oropharyngeal dysphagia [1]. On the other hand, given the high prevalence of GERD, and the difficulty to perform invasive investigations in this group of children, a trial of proton pump inhibitors (PPIs) with careful clinical follow-up is acceptable [1, 26]. PPIs represent the first-line treatment for GERD in children with neurological disability, combined with lifestyle changes, such as the thickening of liquid formula and selection of whey-based, instead of casein-based, enteral formula [1]. Long-term therapeutic regimen with PPIs is often necessary and effective for symptom control and maintenance of remission of esophagitis [27]. If prolonged treatment is required, formal evaluation of GERD and assessment of severity/complications and effectiveness of therapy should be performed. Histamine-2 receptor antagonists are inferior to PPIs for both healing of erosive esophagitis and relief of GERD symptoms [25], and they are not recommended for treating GERD in children with NI. However, up to now no studies evaluating histamine-2 receptor antagonists and PPIs in NI children have been performed. Prokinetic agents, as baclofen, may be considered in uncontrolled GERD, but they are not recommended because of their weak efficacy and side effects [1]. However, they could be considered as rescue therapy before considering surgery. Antireflux surgery should not be considered routinely (for instance, during gastrostomy placement) but only in selected patients, such as those with refractory GERD symptoms, recurrent respiratory illness, and aspiration pneumonia [1, 24]. Primary surgical management of GERD should be performed by Nissen fundoplication. In specific and complex situations (for instance, repeated antireflux surgery), an alternative is represented by the total esophagogastric disconnection [1]. Both procedures seem to have similar efficacy and comparable morbidity. However, esophagogastric disconnection is more invasive and requires longer periods of rehabilitation [28].

- Tip 6: Know how to diagnose and treat refractory constipation

Constipation is significantly more common among children who are tube fed, with a prevalence of 26 to 74% [1, 29, 30]. The causes of constipation include neuromuscular factors, such as intestinal motility disorders, spasticity and hypertonia of the anus and pelvic muscles, hypotonia, and skeletal muscle discoordination; skeletal deformities; prolonged immobility; nutritional factors, such as low fiber and poor fluid intake; drugs. Diagnosis and treatment should be conform to the standard for typically-developing children [1]. Careful history, abdominal, perineal, and if necessary digital rectal examination are recommended [1]. If the diagnosis is uncertain, an abdominal radiograph may be helpful [1]. The initial therapeutic approach involves a fecal disimpaction, using enemas for 3 consecutive days and/or osmotic agents such as polyethylene glycol (1.5 g/kg/day) until stools are liquid and clear, then maintenance therapy with lower dosages (0.8 g/kg/day) [31]. However, use of polyethylene glycol should be suggested with caution (for instance, with lower doses) in children with NI and high risk of aspiration and aspiration-related pneumonia [30]. Due to the chronic use of osmotic agents, children with NI are less responsive to treatment than typically-developing children, so the dose needs to be adjusted [1, 29, 30]. Increasing fiber and fluid intake can be an additional strategy [1, 31].

- Tip 7: Know when to propose a nutritional intervention

One of the most important decisions regarding nutritional management of children with neurological disability is timing the start of enteral nutrition with nasogastric tube feeding or gastrostomy. According to ESPGHAN recommendations [1], enteral nutrition should be considered if: (1) oral intake is insufficient to meet more than 60 to 80% of individual requirements; (2) total feeding time exceeds 3 h per day; (3) there is evidence of inadequate growth or weight gain; (4) there is a fall or a decrease in height velocity; (5) triceps skinfold thickness is consistently less than the fifth centile for age. In addition, enteral nutrition should be considered in the case of severe chewing and swallowing dysfunction, or in the case of aspiration during feeding. One of the most revolutionary statement of the last guidelines is the recommendation to start tube feeding early, even before the development of undernutrition [1]. Attainment of improved growth standards occurs more frequently in children treated early before undernutrition becomes established [32]. Recently factors associated with the need of gastrostomy placement in children with CP have been individuated, such as epilepsy, poor motor function, trunk muscles tone disorder, and male gender [33].

- Tip 8: Be familiar with enteral nutrition: enteral access and feeding regimen

The choice of enteral access depends upon the nutritional and clinical status of the child and the estimated duration of enteral nutrition. Intragastric access is the preferred way as tube insertion is easy and bolus feeds may be utilized [1]. Nutritional support via gastrostomy is indicated in children requiring prolonged enteral tube feeding [1, 34]. Nasogastric tube can be used for a limited time period (< 30 days) only if strictly necessary, for example during waiting time from pre-assessment to tube placement. Gastrostomy can be created surgically (preferably laparoscopic), radiology assisted or more frequently endoscopically (percutaneous endoscopic gastrostomy, PEG). Laparoscopic gastrostomy or laparoscopically-assisted PEG can be safe alternatives in case of severe scoliosis, when the safe placement of PEG is difficult because transillumination of the stomach is not visible. In all those cases in which gastric feeding is contraindicated, such as recurrent vomiting, severe GERD with risk of aspiration, and gastroparesis, jejunal tube feeding is suggested, as an alternative to fundoplication and gastrostomy tube feeding [1, 35]. Jejunal tube feeding is defined as postpyloric feeding through a feeding tube with its tip placed at least 40 cm distally to ligament of Treitz (to prevent retrograde dislodgment of the tube into the stomach) [35]. It can be performed by nasojejunal tube, gastrojejunostomy (for patients who already have a gastrostomy), or jejunostomy. Before jejunal feeding is started, a trial of continuous gastric feeding with a hydrolysed or elemental formula and/or at least one prokinetic drug to promote oral or gastric feeding are recommended [1, 35].

The choice of feeding regimen should be individualized, and based on the type of enteral access, activity level, caloric requirements, and tolerance to feeds [1]. Enteral tube feeding can be delivered as a bolus, intermittently, or continuously. Bolus feeding mimics the physiological endocrine responses to meals with flexible feeding schedules, allows for more freedom, and helps in developing hunger before oral meals. However, it can be not well tolerated in cases of GERD or delayed gastric emptying. Intermittent feeding allows for adjusting the velocity depending on tolerance to feeds. Continuous feeding may be used throughout the day or night and is recommended in the case of poor feed tolerance and jejunal feeding. A combination of overnight continuous feeds, with boluses during the day, may be considered in case of high-caloric needs or poor tolerance to volume [1].

- Tip 9: Know how to choose the best formula

The choice of enteral formula comes from the patient’s age, nutritional requirements and type of enteral access. Breast milk, a standard infant formula, or nutrient-dense infant formula as clinically indicated are recommended before 1 year of age. Standard (1.0 kcal/mL) polymeric age-appropriate formula including fibre is recommended as initial enteral feed after 1 year of age [1, 36]. High-energy density formula (1.5 kcal/mL) including fibre, dietary supplementation with glucose polymers, and/or long-chain triglycerides is suggested for children with increased caloric need or poor tolerance of large volumes of feed. A low-fat, low-calorie (0.75 kcal/mL), high-fibre, and micronutrient-replete formula can be used for children with lower energy needs (for instance immobile children). Whey-based formula may be attempted in cases of GERD, gagging, and retching [1, 37]. This recommendation is mainly based on experts’ opinion. A small study of 10 patients with NI and gastroesophageal reflux were put on a whey-based formula for a 28 h-period, with a subsequent significant reduction in number and duration of reflux episodes [37]. Blenderized or pureed food via gastrostomy should be used with caution because of concerns about nutritional adequacy and safety (such as microbial contamination of enteral tube feeds) [1, 38, 39]. Limited evidence exists on their efficacy in reducing gagging and retching in children after fundoplication [1, 38]. Nonetheless, it has been recently showed that families often choose blenderized food over commercial formulas accessing much of their information online and for several reasons, including the ability to provide foods with natural composition, intimate experiences for parental nurturing, and the benefits that come with family inclusion and mealtime engagement [40].

- Tip 10: Know the benefits of enteral nutrition: efficacy, safety and quality of life

Benefits of enteral nutrition in children with NI encompass better weight gain and nutritional status, ease of feeding, improved medication compliance and reduced symptoms of GERD [36, 41, 42]. A recent retrospective study carried out on 38 NI undernourished children showed a statistically significant increase in triceps skinfold thickness 6 and 12 months after the start of gastrostomy tube feeding with a standard polymeric formula in all children, and a significant increase in body weight and body mass index in 28 of 38 children [36]. Complications associated with gastrostomy and gastrojejunal tubes may involve tube placement (anaesthetic risk, peritonitis, sepsis, gastrointestinal bleeding, invagination) and tube maintenance (dislodgement, obstruction, leakage, tube site infection, granulation tissue) [1, 36, 43]. Minor complications are the most frequent ones [41, 42]. A recent retrospective study carried out on children with CP who presented with a feeding tube-related complaint to two tertiary pediatric emergency departments reported that tube dislodgement was the most common presenting complaint (*n* = 105; 70%). The most common tube type was gastrostomy tube (*n* = 82.5%). Notably, in almost 90% of cases, the feeding tube was successfully replaced in the emergency department, with no need of hospitalization [44]. Children with NI and their caregivers have reduced health-related quality of life (HRQoL), as a consequence of feeding difficulties and gastrointestinal disorders [1]. Children with severe NI are not able to self-report their perceptions of their HRQoL, therefore parent-proxy reports are the only available measures. It has been demonstrated that caregivers of children with NI have worse mental health, higher burnout levels and very poor HRQoL [45]. Enteral nutrition (including home enteral nutrition) showed to positively affect HRQoL of NI children and their caregivers, with significant improvements in physical, psychological and social functioning, mental health, vitality, and in general health perception [46–48].

## Conclusion

The nutritional management of children with NI should encompass thorough evaluation of nutritional status and requirements, prompt nutritional intervention and adequate management of the most frequent gastrointestinal complaints. Main goals are undernutrition prevention, more than reversal, and a satisfying HRQoL of patients and their families. The implementation of these tips in all healthcare settings could potentially improve patient outcomes and reduce morbidity and mortality.

## Data Availability

Unpublished experimental data are not included in this review.

## Abbreviations

CP: Cerebral palsy
DXA: Dual-energy x-ray absorptiometry
GERD: Gastroesophageal reflux disease
GMFCS: Gross Motor Function Classification System
HRQoL: Health-related quality of life
NI: Neurological impairment
PEG: Percutaneous endoscopic gastrostomy
PPIs: Proton pump inhibitors
